# Financial Barriers Decrease Benefits of Interprofessional Collaboration within Integrated Care Programs: Results of a Nationwide Survey

**DOI:** 10.5334/ijic.4649

**Published:** 2020-03-18

**Authors:** Ingrid Gilles, Séverine Schusselé Filliettaz, Peter Berchtold, Isabelle Peytremann-Bridevaux

**Affiliations:** 1 Department of epidemiology and health systems, Centre for primary care and public health, Lausanne University, Lausanne, CH; 2 Forum Managed Care (FMC), Neuageri, CH

**Keywords:** integrated care, interprofessional collaboration, barriers, survey methods, moderated mediations, Switzerland

## Abstract

**Introduction:**

Interprofessional collaboration (IPC) is a key ingredient of integrated care. Nevertheless, IPC benefits remain unclear and its implementation within integrated care initiatives is not straightforward. In this study, we first explored whether IPC was associated with organisational and patient care improvements in Swiss integrated care initiatives; we then investigated the effect of various barriers faced by these initiatives, on these associations.

**Methods:**

Self-reported data from 153 integrated care initiatives included in the Swiss Integrated Care Survey was used. We conducted moderated mediation analyses in which patient care improvements were the outcome, the degree of IPC implementation was the predictor, organisational improvements were the mediator, and professional, patient and financial barriers to integrated care, the moderators.

**Results:**

IPC implementation within integrated care was associated with organisational improvements, which in turn were associated with patient care improvements; this path no longer existed when financial barriers to integrated care were considered.

**Conclusion:**

Organisational improvements should be considered a priority when implementing IPC within integrated care initiatives since patient care improvements due to IPC can be expected mainly when organisational aspects are improved. More importantly, the role of financial barriers should be acknowledged, and actions taken to reduce their impact on integrated care.

## Introduction

Nowadays, chronic diseases and multimorbidity represent considerable burdens and challenges for communities, healthcare systems and individuals. For more than two decades, integrated care initiatives have been considered and implemented throughout Europe and North America as a mean to overcome those challenges [1,2,3,4]. Albeit no consensual definition for integrated care exists [2], many of these initiatives share the following characteristics: patient-centred, promoting patient self-management and autonomy, and based on formal evidence of effectiveness [2]. Moreover, these initiatives aim at restructuring healthcare systems, organisations and services to foster care continuity, coordination, integration, and efficiency [5]. Integrated care initiatives are expected to foster collaboration between various professions [5]; therefore, the involvement of interprofessional teams should represent a key element in such initiatives [6].

Interprofessional collaboration (IPC) occurs:

“when multiple health workers from different professional backgrounds provide comprehensive services by working with patients, their families, carers and communities to deliver the highest quality of care across settings.” [7, p13].

It is considered as an interactional process between healthcare professionals, which includes communication, decision-making and the emergence of shared knowledge and skills [5] to improve both patient and healthcare outcomes [8,9]. Research has shown benefits of IPC for patient care (such as chronic disease care [10]), for patient safety and more globally for the provision of health services [11,12,13]. Besides patient care improvements, IPC is also expected to induce organisational improvements by enhancing care coordination and continuity, promoting equality of status between professionals [14], increasing job satisfaction and engagement [11,12,13,14,15], and creating a healthy workplace [16]. In turn, organisational improvements in care settings has been associated with improved patient care in terms of safety, and fewer adverse events or complications [17,18]. Despite the acknowledgement that IPC is beneficial for both patients and professionals, and despite supportive policy recommendation for its implementation [7,19,20,21,22], IPC remains difficult to operationalize [23,24,25] and is poorly explored when interorganisational aspects are at stake [26], as it is the case in integrated care.

Implementing and maintaining integrated care and IPC initiatives is a complex systemic challenge [27,28] which involves overcoming barriers at three levels: professional, patient and financing [28,29]. Integrated care and IPC both require changes in professional workforce practice as well as more formalized collaborations [30]. More specifically, professionals need to acquire new competences, leadership and management skills, as well as capacities to deal with new roles, clinical activities, responsibilities and decision-making processes, in addition to investing more time in coordination and communication [31]. These adaptations and changes can lead to resistance at the individual and organisational level [32]. At the same time, integrated care requires greater engagement of patients and families in daily care as well as in decision-making processes (e.g. programme planning, care options) [33]. The effectiveness of integrated care initiatives is therefore based on the ability and willingness of chronic patients and family carers to be actively involved in the process. However, financial resources are considered an issue for both the implementation and maintenance of integrated care and IPC. Integrated care stakeholders fear that costs may not be appropriately distributed among structures or professionals involved and expect to face difficulties with the reimbursement of some services such as coordination activities [27,34]. Integrated care initiatives involving IPC are also perceived as costly by professionals, who complain about the lack of adequate resources and remunerations [35].

These barriers can be found worldwide, including rich countries such as Switzerland. In the Swiss context, several financial barriers to the development of integrated care and IPC have been highlighted [36,37]. Even if these barriers have been acknowledge and addressed recently by various initiatives at the federal, cantonal and non-governmental levels [38,39], the Swiss healthcare financing system still strongly favours fee-for-services payments, mono-institutional rates (e.g.: either in-patient or out-patient professionals, not both) and unidirectional care delegation.

**Figure 1:**
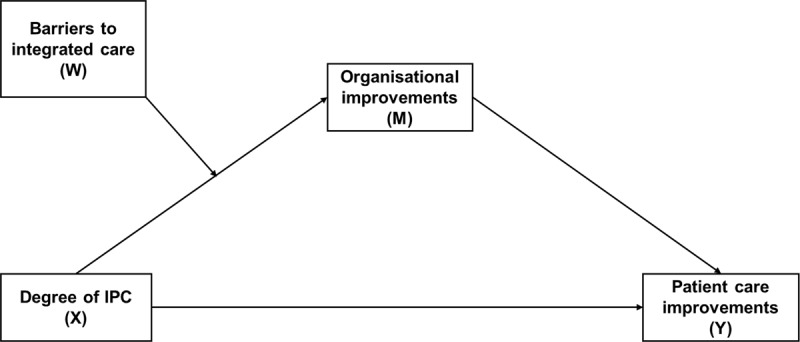
Hypothesized moderated mediation model. X is the predicting variable, Y is the outcome variable, M is the mediator, W is the moderator.

Despite the fact that professional, patient and financial barriers are recognized to undermine the potential positive effect of IPC on patient care within integrated care initiatives, they remain, to our knowledge, scarcely explored [26]. Therefore, the present study aimed at investigating 1) the association between IPC in integrated care initiatives and patient care improvements, via organisational improvements, and 2) the way in which barriers (faced in integrated care initiatives) might condition these associations. First, we hypothesized that IPC within integrated care initiatives would be associated with perceived improvements at the organisational level and consequently at the patient care level (mediation effect, H1). We further hypothesized that this mediation effect would be moderated by professional, patient or financial barriers faced in integrated care initiatives, meaning that the association between IPC and organisational improvements would not be observed if such barriers were present (moderated mediation effect, H2; Figure 1).

## Methods

### Study design and data

In this cross-sectional study, we conducted secondary analyses of self-reported data from the Swiss Survey of Integrated Care (SSIC) [40]. Conducted between July 2015 and July 2016, its aim was to characterize Swiss integrated care initiatives meeting four eligibility criteria: (i) formalization of integrated care principles; (ii) integration of at least two levels of healthcare services (e.g. physician-led primary care, non-physician-led primary care, specialized medical outpatient services, home care services); (iii) integration of at least two different groups of healthcare professionals (e.g. primary care physicians, specialized physicians, nurses (general, specialized or advanced), pharmacists); (iv) initiative continuation during the survey period. Representatives of the 172 eligible integrated care initiatives received an online questionnaire. Data considered for this study are described below.

### Measures

#### The outcome variable: patient care improvements

The SSIC included various aspects of improvement in patient care: patients’ involvement in patient-centred care, informal caregivers’ involvement in care, recognition of informal caregivers’ role, patient satisfaction, patient safety and cost effectiveness. Representatives of integrated care initiatives were asked to state if these aspects had improved in their initiative, using a 4-point Likert scale ranging from 1 = strongly disagree to 4 = strongly agree (good internal consistency for the six items; Cronbach alpha = .84). A mean score ranging from 1 to 4 was computed on these six items, with mean scores close to four indicating the observation of patient care improvements and scores close to one indicating no observation of patient care improvements.

#### The predicting variable: degree of interprofessional collaboration

IPC degree was assessed using 14 items. Thirteen were drawn from the ICARE4EU project [41] and one from previous Swiss research [42]. IPC degree included seven items measuring the extent to which IPC was implemented in the initiative (all relevant professional groups are involved; care providers have a common -professional- language; power positions (e.g. in multi-professional teams) are balanced; attitudes towards the organization, network, model or programme are positive; care providers confidence in each other’s competencies; care providers have sufficient co-operation competencies; interpersonal relationships between care providers are good), and seven items measuring the degree of resistance to the implementation of IPC (care providers are afraid of losing their professional autonomy; different management cultures hinder collaboration; there are barriers for cooperation between medical and non-medical care; there are barriers for information exchange; different working practices of organizations hinder collaboration; over-regulation hinders collaboration; under-regulation hinders collaboration). For each item, representatives of integrated care initiatives were asked to indicate the degree to which the statement corresponded to the reality in practice, using a 4-point Likert scale ranging from 1 = strongly disagree to 4 = strongly agree. Internal consistency for the 14 items was high (Cronbach alpha = .90) and a mean score was computed on the 14 items (scores close to four indicating a high degree of IPC observed in initiatives).

#### The mediator: organisational improvements

The SSIC included four organisational objectives expected to be reached by integrated care initiatives: care coordination; effective cooperation between care providers; adequate competences; professional satisfaction. Representatives were asked to state if these organisational aspects had improved in their initiative, using a 4-points Likert scale ranging from 1 = strongly disagree to 4 = strongly agree. Internal consistency for the five items was acceptable (Cronbach alpha = .70) and a mean score was computed on the four items (scores close to four indicating organisational improvements observed by representatives).

#### The moderators: barriers to integrated care

Eleven barriers to integrated care were considered from the ICARE4EU project [41]: five professional-related barriers (inadequate knowledge/skills of care providers regarding patient involvement; negative attitudes of care providers; inadequate support for care providers; inadequate collaboration between care providers; lack of time of care providers), four patient-related barriers (inadequate patient knowledge/skills in self-management; patient negative attitudes; inadequate support for patients; inadequate support of informal caregivers such as co-care providers) and two financial barriers (inadequate funding (e.g. for implementation of supporting tools); inadequate payment or compensation system). Respondents were asked to state – based on their experience - to what extent these barriers were hampering patient involvement using a 4-points Likert scale ranging from 1 = strongly disagree to 4 = strongly agree. Internal consistency for the three types of barriers was acceptable (all alphas and inter-item correlations >.75); mean scores were computed for each type of barriers (scores close to four indicating presence of barriers).

#### Initiatives’ characteristics

The questionnaire collected additional information about characteristics of the integrated care initiatives: the representatives’ role in the initiatives (11 roles including director/CEO, project manager, nurse, family physician, case manager), the specific targets of the initiatives (patients; family-caregivers; healthcare providers; non-medical care providers; administrative staff), the number of existing supportive interventions for professional collaboration and the number of centred-care interventions, the type and number of professional groups involved (physicians; nurses; paramedical professions; social workers; pharmacists; medical assistants), the total number of professionals in the initiatives, and the geographical area in which the initiatives existed (rural; semi-urban; urban). Using the complete and available information for the initiatives, each one was categorized into one of the following type: mental health and psychiatry; physician networks or health centres; specific groups of patients; transition and coordination; centred on drugs/medications.

### Confounding variables

Several confounding variables were also considered: the amount of supportive actions aiming at fostering collaboration between professionals within the initiative among nine possible components (e.g. training, meetings, quality circle), the amount of patient-centred care components targeted by the initiative among seven possible components (e.g. active involvement of patients in decision-making; supporting patient autonomy in self-care/self-management), the number of professional groups involved among 12 possible categories, and the total number of professionals involved in the initiative.

### Statistical analyses

We first conducted descriptive analyses to characterize the integrated care initiatives. Then, we ran Pearson correlations to assess potential covariations due to confounding variables. Then, we tested our two hypotheses with moderated mediation analyses using linear regressions [43]. This type of analysis is used when an indirect association between three variables is expected to be conditioned by a fourth variable. In other words, moderated mediation analyses enables to show that a mediation process, which is responsible for an effect (i.e. the indirect effect of IPC degree on patient care through organisational improvements), depends on the value of a moderator (i.e. integrated care barriers) [44]. The PROCESS macro [43] we used for these analyses provides an index of moderated mediation [45], and covariates were added to control for confounding effects. A bootstrap procedure was used (95% IC; 5000 samples) to deal with normality issues, and linearity of the residual was assessed with linear regressions. Heteroscedasticity-consistent standard errors’ estimator were applied when the significance of effect was not estimated with bootstrap confidence intervals. Finally, standardized scores were computed and used in the analyses as the questionnaires used different rating scales and first-order interactions were expected.

Since the percentage of missing values was globally low (<3.2%), we performed single imputation using regression models. Descriptive analyses as well as the PROCESS macro for moderated mediation analyses were performed on SPSS Statistics 25; the software GPower [46] was used to test whether the sample size was adequate for estimation analyses. Sample size analyses indicated that a sample of 153 observations was statistically sufficient to reach a power of 0.92 for testing moderated mediation models.

## Results

### Sample characteristics

Of the 172 representatives contacted, 162 returned the survey (94.2% response rate). Responses from nine initiatives were subsequently removed because they were sub-programs of already included initiatives or because they did not target patients. Characteristics of the 153 initiatives included in our analyses are described in detail elsewhere [40].

Briefly: representatives who responded to the questionnaire were mostly directors or project managers (60.2%) or practicing physicians (25.5%). While 60.8% of the initiatives developed integrated care models for specific health conditions (mental health/psychiatry and specific target groups), 18.3% were physician networks or health centres, 15.7% focused on transition and coordination, and 5.2% concerned medicines mainly. All the initiatives targeted patients and 52.9% targeted healthcare professionals (i.e. physicians, nurses, pharmacists, paramedical professions and medical assistants). Among the included initiatives, 86.9% included healthcare professionals and in 65.4% of these initiatives, at least three different professional groups coexisted. Moreover, 60.1% of the initiatives involved a maximum of 10 professionals (irrespectively of their professional group). Also, initiatives mostly included physicians and nurses, whereas paramedical professionals or social workers were involved in less than half of the cases, and pharmacists or medical assistants in one-quarter of the initiatives.

### Moderated-mediation analyses

The results of the preliminary multicollinearity checks (between the predicting variables included in the analyses) are presented in Appendix 1. The three moderated mediation analyses that we then conducted, one per type of barrier, showed the overall index of moderated mediation to be statistically significant for financial barriers (Index = –0.13, Boot 95% CI [–0.23, –0.04]), but not for professional (Index = –0.06, Boot 95% CI [–0.16, 0.02]) or patient-related barriers (Index = –0.05, Boot 95% CI [–0.15, 0.03]), suggesting our hypotheses can only be confirmed for financial barriers (Table 1).

**Table 1:** Regression coefficients for the moderated mediation analysis, with financial barriers as moderator.

Predictor	Outcome of 2-step regression analyses				

	Step 1: Organisational improvements		Step 2: Patient care improvements	

	B	(95%CI)	B	(95%CI)

Number of centred care services	0.06	(–0.10, 0.21)	0.22	(0.06, 0.38)
Number of professionals involved	0.29	(–0.09, 0.15)	–0.21	(–0.31, –0.11)
IPC degree	0.44	(0.27, 0.60)	–0.07	(–0.21, 0.07)
Organisational improvements	–	–	0.51	(0.37, 0.66)
Financial barriers	0.33	(0.16, 0.50)	–	–
IPC degree * financial barriers	–0.25	(–0.41, –0.10)	–	–
R^2^ (%)		21.9***		39.04***
**Conditional indirect effect of IPC implementation on Care improvements due to the initiative**				

	**B**		**95%CI**	

–1 SD below the mean	0.35		(0.20, 0.53)	
Mean	0.22		(0.13, 0.34)	
+1 SD above the mean	0.09		(–0.01, 0.21)	
Moderated mediation index (with Boot 95% CI)	–0.13 (–0.23, –0.04)			

*Note*: Scores are standardised; IPC degree * financial barriers = interaction between IPC degree and financial barriers.

**Figure 2:**
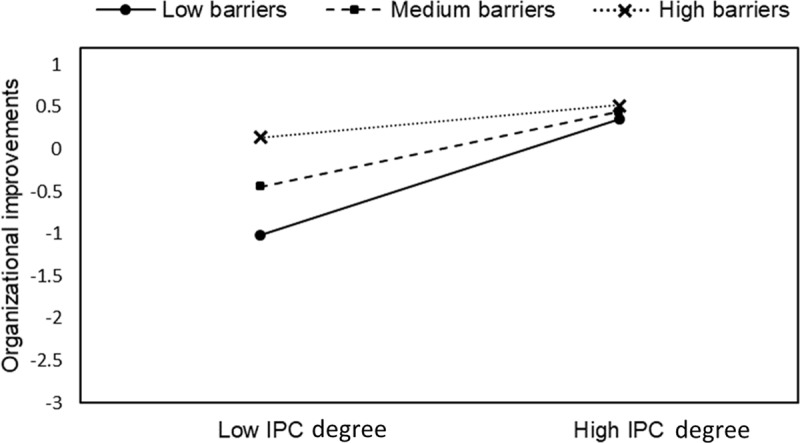
Interaction of IPC degree and financial barriers on organisational improvements. Note: Low IPC = 1 SD below the mean; High IPC = +SD above the mean.

Indeed, analyses revealed an indirect effect of IPC degree on patient care improvement through organisational improvements: a high score of IPC degree was actually associated statistically with an increase of the organisational improvements score (B = 0.44, 95% CI [0.27, 0.60]), which was statistically associated with an increase of the patient care improvements’ score (B = 0.51, 95% CI [0.37, 0.66]). This, in addition to the fact that the direct effect of IPC degree on patient care improvements was not significant (B = –0.07, 95% CI [–0.21, 0.07]) confirmed our mediation hypothesis (H1). Moreover, as hypothesized, the indirect effect of IPC degree on patient care improvements was conditional on the presence of reported financial barriers (see details in Table 1). In fact, the indirect effect was statistically significant when respondents reported low or medium financial barriers (mean or –1SD below the mean) but not when they reported high financial barriers (+1 SD above the mean). More specifically, financial barriers moderated the association between the degree of IPC and organisational improvements (B = –0.25, 95% CI [–0.41, –0.10]), suggesting that financial barriers faced by integrated care initiatives hindered the association between IPC degree and organisational improvements (Figure 2).

Detailed results of the professional and patient-related barriers are available in Appendix 2.

## Discussion

The results of this study confirm our moderated mediation hypotheses for financial barriers only. This suggests that IPC degree within integrated care initiatives was associated with patient care improvements through organisational improvements. However, this was less observed in initiatives facing financial barriers for the implementation of integrated care.

To our knowledge, this is the first study investigating whether the association between the degree of IPC in integrated care and patient care improvements is mediated by organisational improvements. In fact, our results complement the current literature about the impact of IPC on job satisfaction and well-being [11], suggesting that organisational improvements are necessary for IPC to improve patient care in integrated care initiatives. In other words, IPC interventions should adopt a systemic approach to achieve patient care improvements. This is in line with conceptual models considering care outcomes as products of interacting elements. For example, the Chronic Care Model [47] promotes productive interactions between prepared, proactive practice teams and informed, active patients, in addition to organisational adaptations (i.e. a high level of professional engagement, development of new skills and responsibilities), to bring benefits to patients [48]. Also, De Savigny and Adam [29] consider six important building blocks when strengthening the health system (i.e. leadership/governance, service delivery, human resources, information, financing, medical products, vaccines and technologies, and people) and advocate for a better understanding of the “nature of relationships” among building blocks.

We made the hypothesis that barriers faced by integrated care initiatives could hinder IPC, and found that financial barriers (such as inappropriate patient reimbursements or inadequate funding as measured in our questionnaire) affected the degree to which IPC was implemented within integrated care initiatives. In such contexts, the existence of financial barriers has already been highlighted in the literature. However, they have mostly been described as covert than as major barriers [35]. For example, in a recent review on professionals’ experiences with IPC in primary care, financial barriers were not cited as such by professionals in any of the 21 included studies [49]. The difference between our results and the latter could have three explanations. First, most studies included in the above-mentioned review used qualitative methods and financial barriers were not directly measured. As the latter had to emerge from professionals’ discourse, it is likely that financial issues were embedded in more complex representations of factors hindering IPC. For example, financial issues could have been assimilated to organisational barriers in professionals’ representations because a lack of financial resources leads to increased workloads or coordination issues. Second, in our study, the majority of respondents were directors or project managers and not professionals directly involved in patient care. As shown in Germany, managers are more likely to explicitly talk about administrative and other cost issues [50]. Also, discrepancies between managers and professionals in their perception of the effect of financial aspects on IPC have been described. Indeed, when managers supported the idea of financial solutions (i.e. a shared budget) favouring care coordination and collaboration, professionals considered IPC as requiring a high staff commitment [51]. This suggests that financial barriers of both integrated care and IPC are mainly experienced at the managers’ level, which is important information considering they are leading the implementation and maintenance of integrated care initiatives.

The question of financial resources remains central when considering IPC within integrated care initiatives. Even though implementing such initiatives is costly, initial financial investment is key for the success of integrated care initiatives [6]. However, this initial financial effort may be prohibitive for many integrated care managers [52]. Also, even if IPC is expected to be cost-effective for both patients and the healthcare system [11], cost-saving evidence and the time lapse needed for managers to observe such benefits remains less obvious.

There is a clear need for innovation in the financing of integrated care initiatives [53]. Our results suggest targeting organisational aspects, for instance, supporting the development of professionals’ collaborative competences or facilitating coordination and cooperation between actors within initiatives. In Switzerland, the need for innovative financing models has also been acknowledge by healthcare stakeholders [36,54]. Some efforts have been made to promote the uniformization of funding between the ambulatory and hospital sectors (monistic funding), but until now, without concrete changes [55]. Nevertheless, the fee-for-services payment system and high health insurance premiums remain major barriers to the further development of integrated and coordinated care in Switzerland [56].

While interpreting these results, the following limitations need to be considered. First, the operational definition of integrated care used here may be discussed [40]. Nevertheless, it was developed after gathering criterion from the literature and discussing with integrated care experts. Second, the data collected was self-reported by representatives of the initiatives, which may lead to response bias. Third, the cross-sectional study design precludes causality ascertainment. Notwithstanding these limitations, we do think that the results of this study will benefit the integrated care community and help further explore financial allocation models.

## Conclusion

This study suggests that IPC implementation within integrated care initiatives leads to organisational improvements, which then benefit patient care. Additionally, it shows that financial barriers interfere with that process. Studies evaluating the impact of IPC within integrated care initiatives should not only target patient care improvements but should also consider organisational ones. More importantly, the role of financial barriers to the development of integrated care should be acknowledged and actions taken to reduce them both at the implementation and at the maintenance stages.

## Additional Files

The additional files for this article can be found as follows:

10.5334/ijic.4649.s1 Appendix A. Descriptive statistics and Pearson’s correlations for confounders and variables of interest.

10.5334/ijic.4649.s2 Appendix 2. Regression coefficients of moderated mediation analysis with professional- and patient-related barriers as moderator.
