# Supplementary material for: Financial Barriers Decrease Benefits of Interprofessional Collaboration within Integrated Care Programs: Results of a Nationwide Survey
Source: Int J Integr Care. 2020 Mar 18;20(1):10. doi: 10.5334/ijic.4649 (PMC7101009; doi:10.5334/ijic.4649)
Supplement: Appendix A. — Descriptive statistics and Pearson’s correlations for confounders and variables of interest. [file ijic-20-1-4649-s1.pdf]

Appendix 1

Descriptive statistics and Pearson's correlations for confounders and variables of interest

Variables	Mean	SD	Variables								
			1	2	3	4	5	6	7	8	9
Confounders											
1. Number of supportive interventions for professionals	4.8	2.3	--								
2. Number of Centred-care interventions	4.0	2.0	.38**	--							
3. Number of professionals involved	41.5	121.6	-.04	-.23**	--						
4. Number of professional groups	3.2	1.3	.36*	.30**	.02	--					
Variables of interest											
5. IPC degree	3.1	0.5	.00	.20*	-.13	-.08	--				
6. Financial barriers	2.8	1.0	.18*	.09	.05	.21**	-.38**	--			
7. Professional-related barriers	2.3	0.7	.06	-.02	.07	.09	-.49**	.52**	--		
8. Patient-related barriers	2.3	0.7	-.00	-.00	.04	.13	-.38**	.46**	.76**	--	
9. Organisational improvements	3.6	0.4	.17*	.14	-.03	.09	.32**	.09	-.10	-.06	--
Outcome											
10. Care improvements in the initiative	3.4	0.5	.11	.33**	-.27*	.07	.17*	.10	-.03	-.02	.53**

* $p < 0.05$; ** $p < 0.01$.

Note: The three types of barriers (financial, professional-related and patient-related) have not been entered in the same moderated mediation analysis, however, we present them on the same correlation table to avoid redundant information.
