# Supplementary material for: Financial Barriers Decrease Benefits of Interprofessional Collaboration within Integrated Care Programs: Results of a Nationwide Survey
Source: Int J Integr Care. 2020 Mar 18;20(1):10. doi: 10.5334/ijic.4649 (PMC7101009; doi:10.5334/ijic.4649)
Supplement: Appendix 2. — Regression coefficients of moderated mediation analysis with professional- and patient-related barriers as moderator. [file ijic-20-1-4649-s2.pdf]

Appendix 2

A. Regression coefficients of moderated mediation analysis with professional-related barriers as moderator.

	Outcome of 2-step regression analyses			
	Step 1 : Organisational improvements		Step 2 : Patient care improvements	
Predictor	B	(95%CI)	B	(95%CI)
Number of centred care services	0.10	(-0.06, 0.25)	0.22	(0.06, 0.38)
Number of professionals involved	0.04	(-0.10, 0.19)	-0.21	(-0.31, -0.11)
IPC degree	0.36	(0.15, 0.57)	-0.07	(-0.21, 0.07)
Organisational improvements	--	--	0.51	(0.37, 0.66)
Professional-related barriers	0.11	(-0.09, 0.30)	--	--
IPC degree * Professional-related barriers	-0.11	(-0.28, 0.06)	--	--
R^2 (%)	12.6		39.04	
Conditional indirect effect of IPC implementation on Care improvements due to the initiative				
	B		(95%CI)	
-1 SD below the mean	0.24		(0.10, 0.42)	
Mean	0.19		(0.08, 0.31)	
+1 SD above the mean	0.13		(-0.01, 0.25)	
Moderated mediation index (with Boot 95% CI)	-0.06 (-0.16, 0.02)			

B. Regression coefficients of moderated mediation analysis with patient-related barriers as moderator.

	Outcome of 2-step regression analyses			
	Step 1 : Organisational improvements		Step 2 : Patient care improvements	
Predictor	B	(95%CI)	B	(95%CI)
Number of centred care services	0.09	(-0.06, 0.25)	0.22	(0.06, 0.38)
Number of professionals involved	0.04	(-0.10, 0.18)	-0.21	(-0.31, -0.11)
IPC degree	0.34	(0.14, 0.55)	-0.07	(-0.21, 0.07)
Organisational improvements	--	--	0.51	(0.37, 0.66)
Patient-related barriers	0.09	(-0.10, 0.26)	--	--
IPC degree * Patient-related barriers	-0.10	(-0.27, 0.08)	--	--
R^2 (%)	12.4		39.04	
Conditional indirect effect of IPC implementation on Care improvements due to the initiative				
	B		(95%CI)	
-1 SD below the mean	0.22		(0.09, 0.39)	
Mean	0.18		(0.08, 0.30)	
+1 SD above the mean	0.13		(-0.01, 0.25)	
Moderated mediation index (with Boot 95% CI)	-0.05 (-0.15, 0.03)			

Note: Scores are standardised; IPC degree * Patient-related barriers = interaction between IPC degree and patient-related barriers.
